# Ensuring communication redundancy and establishing a telementoring system for robotic telesurgery using multiple communication lines

**DOI:** 10.1007/s11701-023-01792-8

**Published:** 2024-01-11

**Authors:** Yusuke Wakasa, Kenichi Hakamada, Hajime Morohashi, Takahiro Kanno, Kotaro Tadano, Kenji Kawashima, Yuma Ebihara, Eiji Oki, Satoshi Hirano, Masaki Mori

**Affiliations:** 1 Committee for Promotion of Remote Surgery Implementation, Japan Surgical Society, Tokyo, Japan; 2 Department of Gastroenterological Surgery, Hirosaki University Graduate School of Medicine, 5 Zaifu-Cho Hirosaki, Aomori, 036-8562 Japan; 3 RIVERFIELD Inc, Tokyo, Japan; 4 Department of Information Physics and Computing School of Information Science and Technology, The University of Tokyo, Tokyo, Japan; 5 Department of Gastroenterological Surgery II, Faculty of Medicine, Hokkaido University, Sapporo, Japan; 6 Department of Surgery and Science, Kyushu University, Fukuoka, Japan; 7 Tokai University School of Medicine, Isehara, Japan

**Keywords:** Multiple communication lines, Robotic telesurgery, Telementoring system

## Abstract

Assuring communication redundancy during the interruption and establishing appropriate teaching environments for local surgeons are essential to making robotic telesurgery mainstream. This study analyzes robotic telesurgery with telementoring using standard domestic telecommunication carriers. Can multiple carriers guarantee redundancy with interruptions? Three commercial optical fiber lines connected Hirosaki University and Mutsu General Hospitals, 150 km apart. Using Riverfield, Inc. equipment, Hirosaki had a cockpit, while both Mutsu used both a cockpit and a surgeon’s console. Experts provided telementoring evaluating 14 trainees, using objective indices for operation time and errors. Subjective questionnaires addressed image quality and surgical operability. Eighteen participants performed telesurgery using combined lines from two/three telecommunication carriers. Manipulation: over 30 min, lines were cut and restored every three minutes per task. Subjects were to press a switch when noticing image quality or operability changes. Mean time to task completion was 1510 (1186–1960) seconds: local surgeons alone and 1600 (1152–2296) seconds for those under remote instructor supervision, including expert intervention time. There was no significant difference (*p* = 0.86). The mean error count was 0.92 (0–3) for local surgeons and 0.42 (0–2) with remote instructors. Image quality and operability questionnaires found no significant differences. Results communication companies A, B, and C: the A/B combination incurred 0.17 (0–1) presses of the environment change switch, B/C had 0, and C/A received 0.67 (0–3), showing no significant difference among provider combinations. Combining multiple communication lines guarantees communication redundancy and enables robotic telementoring with enhanced communication security.

**Supplementary Information:**

The online version contains supplementary material available at 10.1007/s11701-023-01792-8.

## Introduction

In Japan, robotic surgery for total prostatectomy was covered by insurance in 2012, and then gradually robotic surgery began to spread nationwide, centered on the da Vinci^®^ (Intuitive Surgical) equipment. The indication for robotic surgery has been expanded to various fields, such as gastrointestinal surgery, respiratory surgery, and gynecology, due to further insurance revisions in April 2018 and April 2020, so robotic surgery is becoming more common in high-volume centers and general city hospitals. In the past decade, Japan has become one of the world's leading robotic nations, and the role of robotic surgery in daily medical practice is increasing year by year.

In recent years, expectations for remote surgical support with the guidance (telementoring) of highly skilled physicians have been increasing against the backdrop of advances in surgical robotics and high-speed, large-volume communication technology [1–3]. We have conducted several studies involving demonstrations with the goal of realizing telesurgery. To that end, guidelines for telesurgery were developed in Japan in April of 2022. The April 2022 guidelines for telesurgery in Japan are based on next-generation robotic equipment, Riverfield's Saroa^TM^ and Japanese commercial communication lines. The delay time was shown to be acceptable for telesurgery [4–8]. In the same year, after conducting preliminary experiments in Kobe, Japan, a telesurgery system was also constructed between hospitals in Hirosaki and Mutsu using hinotori^TM^ from Medicaroid Corporation of Japan, showing that multiple domestic robots can achieve successful telesurgery using ordinary Japanese communication lines [9–11].

However, there are still multiple issues to be addressed to realize telesurgery. First, there is the control of communication delay in robot operation and the transmission and reception of good image and picture quality. These issues all can be overcome with the current surgical robot technology, communication technology, and information processing technology. On the other hand, issues to be solved in the future include reliability concerns when communication is interrupted, economic efficiency, and ethical issues. Communication interruptions during surgical support for inexperienced surgeons from a remote location, in particular, could result in patient fatalities. Communication failures can occur suddenly for various reasons, including natural disasters, but until now, only NTT East's lines have been considered. To strengthen communication reliability, it is necessary to incorporate various telecommunication carriers. The purpose of this study is to construct a telementoring system using the communication lines of multiple Japanese telecommunication carriers and to examine whether it is then possible to guarantee redundancy by using multiple carriers simultaneously in the event of communication interruptions.

## Material and methods

### Network connections

Hirosaki University Hospital (Hirosaki, Aomori, Japan) and Mutsu General Hospital (Mutsu, Aomori, Japan), 150 km north of Hirosaki, were connected by commercial optical fiber. The optical fiber network used communication lines was provided by three companies: SoftBank Corporation (Tokyo, Japan), NTT East Corporation (Tokyo, Japan), and Tohoku Intelligent Telecommunication Corporation (Miyagi, Japan) Two types of lines were provided: guaranteed lines (maximum speed 100 Mbps) and best-effort lines (maximum speed 1 Gbps). An IP-VPN (Internet Protocol-Virtual Private Network) was also constructed using these lines. The guaranteed lines were adjustable according to the subscriber's secured bandwidth usage needs. A quality of service guarantee system and 24/7 support were standard features. Best-effort lines feature transmission speeds of up to 1 Gbps and are relatively inexpensive, but actual speeds vary depending on line congestion. The communication information is compressed and decompressed using Soliton's encoder: Zao-SH, and decoder: Zao-View (Soliton Systems Corporation, Tokyo, Japan). The encoder and decoder used in this study are Real-time Automatic Speed Control Based on the Waterway Model (RASCOW™), a high compression technology that enables ultra-short delay video transmission and has been applied to ultra-short delay live broadcasting.

### Surgical robot system

The robot was a pneumatically driven robot system, Saroa^TM^, from Riverfield Inc (Tokyo, Japan) [12]. The cockpit was installed at Hirosaki University and Mutsu General Hospital, and the surgeon console was installed only at Mutsu General Hospital.

### Telesurgery using the dual console education system (Experiment 1)

**Fig. 1:**
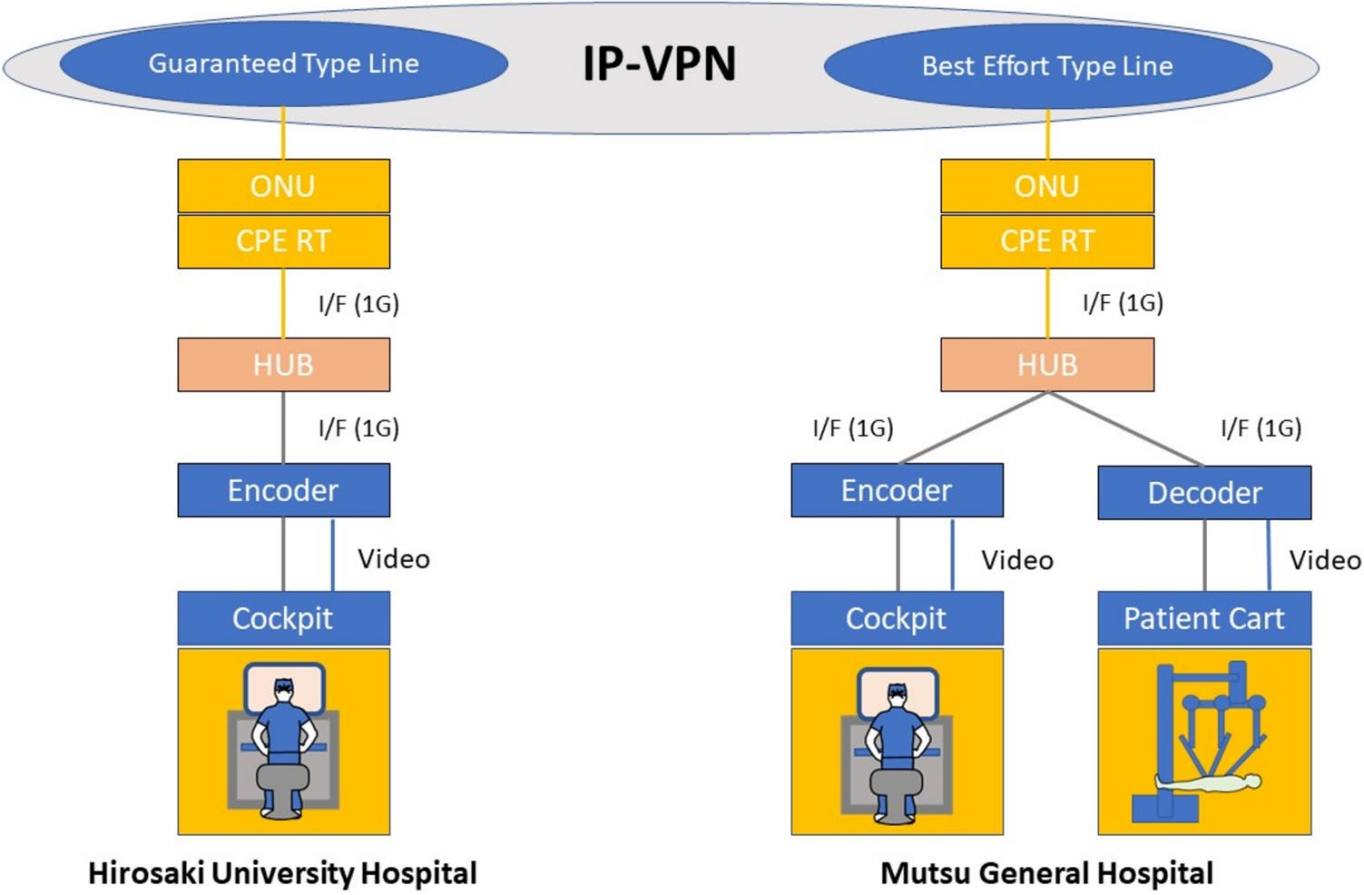
Network system of SoftBank Co. OUN: Optic network unit, CPE RT: Customer premises equipment remote terminal

**Fig. 2:**
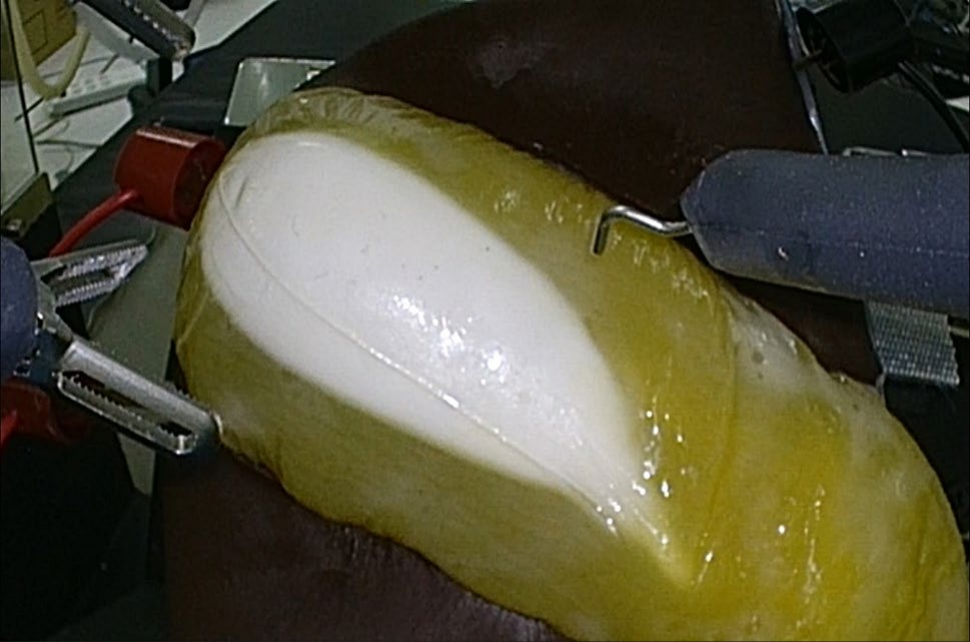
Artificial organ model, FASOTEC gallbladder model

Experiments were conducted for the purpose of remote surgical guidance (Experiment 1) and for communication redundancy by utilizing the combined services of multiple telecommunication companies (Experiment 2) (Table 1). In Experiment 1, telementoring was provided to 14 trainee physicians in the local environment through the intervention of a remote instructor by four specialists in the remote environment. The communication line was provided by SoftBank (Fig. 1). First, the practitioners performed surgery on the FASOTEC gallbladder model in the local environment. The main raw material of FASOTEC gallbladder models is PVA (PolyVinyl Alcohol), which is used to form each component whether it be a liver, a gallbladder, or vessels, for example, and then each one is fashioned into the final shape. FASOTEC Corporation, located in Chiba Prefecture, is responsible for the development, design, and sales of the product, while Sunarrow Corporation, located in Tokyo, is responsible for the manufacturing (Fig. 2). Subsequently, as a single trial, until the end the of resection, the practitioner performed surgery on the gallbladder model while receiving telementoring from a specialist in the remote environment. Surgical guidance from a remote location was provided using the swapping and annotation functions developed by Riverfield. The swapping function is a function that connects two surgeons’ consoles, one at the home site and the other at a remote site and allows a trainee surgeon to change places with an instructor when performing a difficult operation. The annotation function is a function that displays local surgical images on a touch panel display and allows the instructor to instruct the trainee while drawing lines and arrows. The items to be evaluated were communication delays of robotic operation, achievement and error rates, and various operability scores using Image Quality Score, mSUS, and Robot Usability Score, which all evaluate the type of image quality degradation caused by changes in the communication environment and the consequent impact on the procedure, clarity, stereopsis, completeness, continuity, and impact on technique scores. Higher scores on a 5-point scale indicated no degradation in image quality and no impact on the procedure (Supplementary Table 1). The total score for each item was used in the evaluation. The “mSUS” used in the evaluation was a modified version of the System Usability Scale (SUS) proposed by Brooke [13]. Nine items were rated on a 5-point scale, and the total score was calculated. The higher the score, the higher the evaluation, so a low score indicates a low evaluation (Supplementary Table 2). Effectiveness is shown in Supplementary Table 3. Robot operability was evaluated on a 5-point scale to assess ease of operation. For this, the evaluation method reported by Sharker et al. was modified to evaluate the accuracy, number of errors, and time required to complete the procedure [14]. The number of errors was scored on a scale of 0 to 6 for each ligation procedure, and accuracy was scored on a scale of 0 to 6. YES = 1 and NO = 0 for each of the six items.

**Table 1:** Summary of the experiments

Experiment	1	2
Purpose	Remote surgical guidance	Communication redundancy by combining multiple telecommunication companies
Objectives	4 specialists and 14 trainee physicians	3 specialists and 15 trainee physicians
Communication Lines	SoftBank Corporation	SoftBank Corporation, NTT East Corporation, Tohoku Intelligent Telecommunication Corporation
Methods	The practitioner performed surgery on the gallbladder model while receiving telementoring from a specialist in a remote environment	The two communication lines are repeatedly shut down and restarted during the operation of the gallbladder and stomach models, and the switch is flipped when abnormalities in the images or procedures are noticed
Assessment Items	Image Quality Score, mSUS, Robot Usability Score	Image Quality Score, Circuit Interruption Questionnaire

### Verification of communication redundancy using multiple communication lines (Experiment 2)

**Fig. 3:**
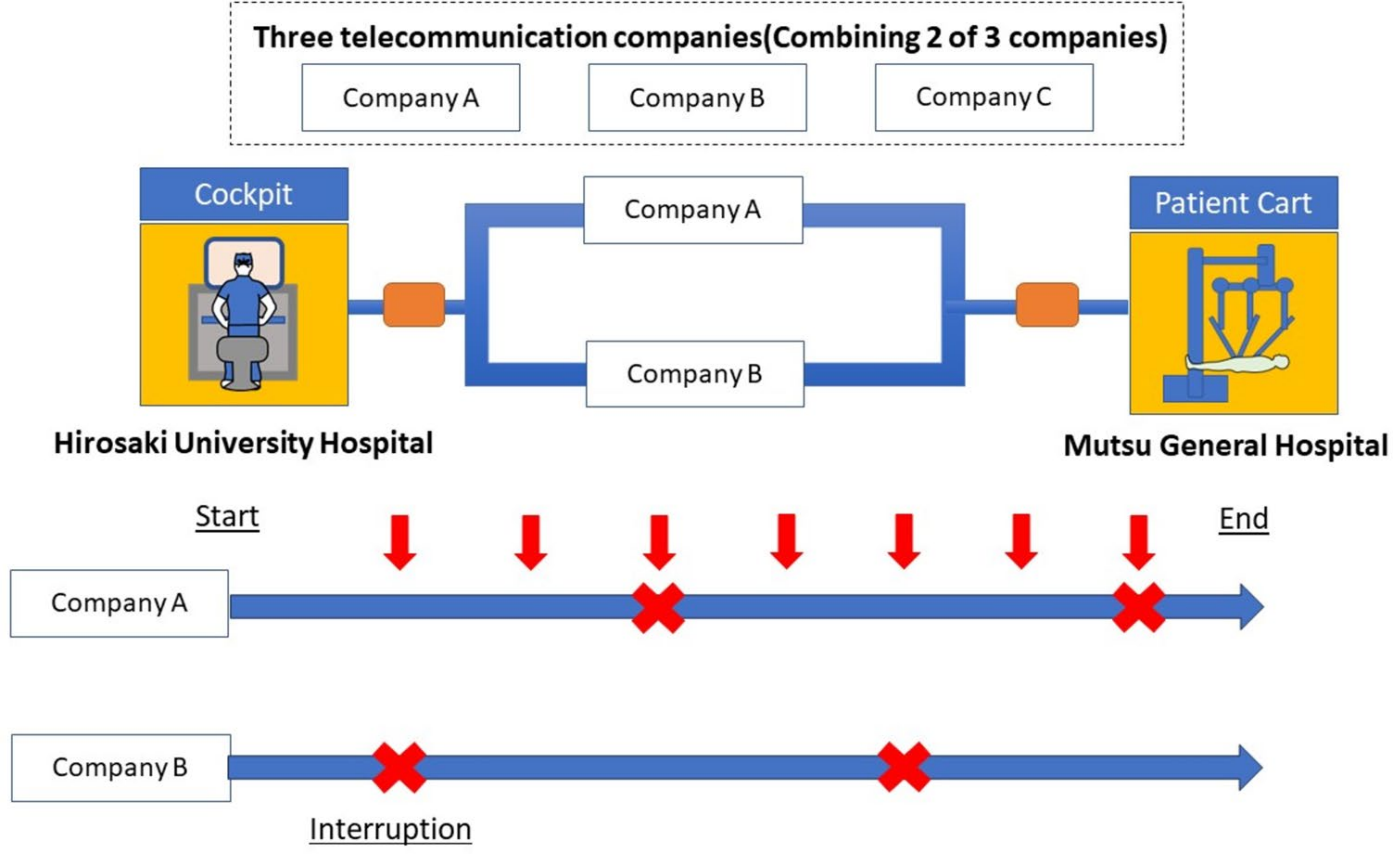
Overview of Experiment 2

In Experiment 2, the subjects were three gastroenterological surgeons at Hirosaki University Hospital who were experts in robotic surgery and 15 surgeons with limited experience in robotic surgery. There were no conflicts of interest with the robotics or telecommunication companies involved in the experiment. The communication environment was set up so that two of the three telecommunication companies (SoftBank Corporation [Tokyo, Japan**]**, NTT East Corporation [Tokyo, Japan**]**, and Tohoku Intelligent Telecommunication Corporation [Miyagi, Japan**]**) were connected to each other, with six participants testing each combination (Company A/Company B, Company B/C, and Company C/A), comprising the total of 18 participants. One of the two communication lines used between the start and end of the experiment was set up to randomly shut down and restart repeatedly (Fig. 3). When a disturbance to the image was detected during the procedure, a switch at the foot of the patient's bedside was pressed to signal the operator. Cholecystectomy or gastrectomy was performed on the FASOTEC gallbladder and stomach models. The evaluation items were the Image Quality Score and the Circuit Interruption Questionnaire. The Circuit Interruption Communication Questionnaire was used to check whether changes in the surgical environment were noticeable, so those who noticed changes were asked about image quality and robotic operability (Supplementary Table 4). Next, participants rated each change on a 5-point scale for inhibition of surgical maneuvering and the likelihood of completing the procedure, with higher scores indicating that surgical maneuvering was unaffected.

### Surgical techniques for artificial organ models

The right arm of the Saroa was dissected using electrocautery with monopolar scissors forceps and the left with bipolar windowed forceps. After recognizing the gallbladder duct and gallbladder artery, the gallbladder model was ligated and dissected proximally and distally. The gallbladder artery was ligated and dissected with a 2–0 silk thread. The gallbladder was then detached from the liver bed to complete the process. In the gastric model, the omental bursa was opened, the left gastroepiploic artery was dissected, and then an incision was made on the right side to dissect the right gastroepiploic artery. The right gastric artery was then dissected, and gastrectomy was performed by a local assistant, followed by the dissection of the left gastric artery.

### Statistical analysis

EZR was used for statistical analysis. Normality tests were performed with the Kolmogorov–Smirnov test, and if normality was not rejected, a paired t-test was used. If normality was rejected, the Wilcoxon signed rank sum test was used. Statistical significance was determined at *P* < 0.05.

## Results

### Telesurgery using the Dual console education system

The mean time for cholecystectomy was 1510 (1186–1960) seconds for local surgeons only and 1336 (866–2127) seconds when remote instructor intervention was needed (excluding instructor operation time), with no significant difference between the two (*p* = 0.14). Under remote instructor intervention (including instructor intervention time), the mean error count was 1600 (1152–2296) seconds, but there was no significant difference (*p* = 0.86). The mean number of errors was 0.92 (0–3) for the local surgeons and 0.42 (0–2) for the remote instructors (Table 2). No apparent communication failures occurred during the remote guiding trials.

**Table 2:** Operation time and number of errors under telementoring. Values are averages (ranges). * p < 0.05

	Local	Remote	*p* value
Operation time (sec)	1510 (1186–1960)	1336 (866–2127)	0.143
Error (times)	0.92 (0–3)	0.42 (0–2)	0.19
Operation time included supervising (sec)	1510 (1186–1960)	1600 (1152–2296)	0.862

### Image quality score

The mean values for mSUS were 20.1 (14–25) for local surgeons and 18.0 (14–22) for remote instructors, not significantly different (*p* = 0.48). mSUS was 25.0 (18–38) for local surgeons and 28.3 (23–39) for remote instructors, no significant difference between the two (*p* = 0.95)(Table 3).

**Table 3:** Image and operability scores under telementoring. Values are averages (ranges). * p < 0.05

	Supervisor	Operator	*p* value
mSUS (Max 45 points)	28.3 (23–39)	25 (18–38)	0.952
Robot Usability Score (Max 40 points)	30 (22–36)	30.3 (18–33)	0.391
Image Quality Score (Max 25 points)	18 (14–22)	20.1 (14–25)	0.427

### Robot usability score

Local surgeons averaged 30.3 (18–33) while it was 30 (22–36) for remote instructors, with no significant difference between them (*p* = 0.39) (Table 3).

### Verification of communication redundancy using multiple communication lines

#### Line switching

The three companies were assigned aliases as Company A, Company B, and Company C. The Company A/B combination, hereafter called “Company A/B,” had 0.17 (0–1) presses of the communication environment change switch, while the Company B/C duo, hereafter abbreviated “Company B/C,” had 0 and the Company C/A iteration, hereafter “Company C/A,” had 0.67 (0–3) presses, yielding no significant differences among the combinations (Table 4). The timing of pressing the switch did not coincide with the timing of the actual environmental changes, even for the subjects who claimed to have noticed changes.

**Table 4:** Subject cues when switching lines. Values are averages (ranges). * p < 0.05

	A/B	B/C	C/A	*p* value
Subject Cues [times]	0.17 (0–1)	0	0.67 (0–3)	0.304

### Image quality score

The Image Quality Score was 19.7 (11–25) for Company A/B, 19.7 (17–23) for Company B/C, and 20.0 (18–23) for Company C/A, showing no significant difference (*p* = 0.87) (Table 5).

**Table 5:** Image Quality Score of the line switching experiment. Values are averages (ranges). * p < 0.05

	A/B	B/C	C/A	*p* value
Image Quality Score	19.7 (11–25)	19.7 (17–23)	20.0 (18–23)	0.869

### Questionnaire for changing line conditions

Subjects noticed changes in the communication environment in Question 1 as follows: Company A/C Company B 0.17 (0–1), Company B/C Company 0 (0–0), and Company C/C Company A 0.17 (0–1). The scores for question 2 regarding inhibition of surgical manipulation were 4.83 (4–5) for Company A/B, 4.67 (3–5) for Company B/C, and 4.83 (4–5) for Company C/A, with no significant differences (*p* = 0.99). The scores for question 3 regarding the likelihood of completing the surgery were not significantly different (*p* = 0.62), with Company A/B 4.33 (3–5), Company B/C 4.67 (4–5), and Company C/A 4.33 (4–5) (Table 6).

**Table 6:** Questionnaire for changing line conditions. Values are averages (ranges). *p < 0.05

	A/B	B/C	C/A	*p* value
Question 1 (Number of people who noticed the line change)	0.17 (0–1)	0	0.17 (0–1)	0.588
Question 2	4.83 (4–5)	4.67 (3–5)	4.83 (4–5)	0.990
Question 3	4.33 (3–5)	4.67 (4–5)	4.33 (4–5)	0.624

## Discussion

In this study, we showed that a telementoring system can be constructed using SoftBank lines and Saroa^TM^, and that a redundant configuration using lines from several different telecommunication carriers is possible in the event of communication interruption.

Robot-assisted surgery is an endoscopic surgical procedure, so to speak, in which the surgeon sits in a pilot's seat at a distance from the patient's operating table and remotely operates a surgical robot while viewing images of the operating field, thus performing endoscopic surgery at a slight distance in the operating room. In recent years, the development of information and communication technology and the development of telesurgery-compatible robots have made it possible to perform teleoperations by placing the operating robot and the patient's operating table in separate facilities some distance from each other and connecting them via a high-speed, large-capacity communication network.

Telementoring is a type of telesurgery in which the tele-mentor provides verbal and graphical guidance to a local doctor, as well as remote surgical support where the telementor operates the robot remotely to assist the local doctor in surgery; full telesurgery is when the telementor performs the surgery alone. To summarize, the three concepts are telementoring, remote surgical support, and full telesurgery. [15, 16]. While telementoring is already available, the Japanese guidelines for online medical care do not recognize full telesurgery. Remote surgical support, on the other hand, is a format that is recognized by the Japanese online practice guidelines and is feasible. The establishment of a remote surgical education system is expected to help solve social issues such as regional disparities in medical care and the shortage of surgeons [17]. Surgeons working in rural areas can obtain surgical support from skilled physicians, and young surgeons can receive continuous surgical guidance from their mentors at the main facility.

This study showed that telementoring using swapping and annotation functions could be performed smoothly without delay or image quality distortion. There was no difference in operative time and the number of errors, including instruction in the local environment and in the remote environment for either the surgeon receiving the instruction or the instructor providing the instruction. Conversely, with relatively difficult surgeries in the field of gastrointestinal surgery, such as gastrectomy and rectal resection, it is expected to be difficult for inexperienced surgeons to complete the operation in the same amount of time as the instructor, but the use of the swapping function on the instructor's side is expected to contribute to shortening the operation time. This point is also considered to be very useful in ensuring the quality of the operation for the local surgeon who is receiving instruction.

Robotic surgery is becoming popular as a technology that overcomes movement limitations, a weak point of traditional endoscopic surgery, and thus enables precise, minimally invasive surgery [18–20]. On the other hand, unlike its current status, robotic surgery was originally developed for the purpose of telesurgery [21]. In 2001, the world's first remote laparoscopic cholecystectomy was performed using Zeus, connecting New York and Strasbourg, France, 7000 km away, via a dedicated communication line [22]. In 2003, 22 cases of telesurgery were performed in Canada [16]. All were successful, but these projects came to an end when Zeus discontinued production due to a corporate merger.

Subsequently, in the U.S., a demonstration experiment was conducted using the da Vinci Surgical System with an internet connection [23, 24], and in Japan, a demonstration experiment was conducted using a proprietary robot and connecting facilities in Japan and overseas with a dedicated line. However, the commercial and internet lines used had insufficient integrity and security, the communication lines were extremely expensive, and the information processing technology could not reduce the transmission delay time to a level that would allow clinical applications, all of which were decisive factors that led to a long hiatus, suspending new telesurgery research for a long period of time.

It is very important to construct a system that can safely continue surgery in the event of communication problems when providing education to remote areas. We have conducted telesurgery experiments using an NTT East line and demonstrated that telesurgery could be performed safely without interruption or delay due to serious communication failures, and in this verification, we showed that the same safe telesurgery is possible using a SoftBank line. However, it is difficult to guarantee sufficient communication redundancy when communication is interrupted during telesurgery if only a single line is used. In our previous experiments, by providing two types of communication lines, it was possible to construct a system in which communication interruptions during robotic telesurgery would not affect the images during the surgery or the continuity of the robot operation [8]. In this experiment, robotic telesurgery was performed in an environment where communication lines of multiple telecommunication companies were used simultaneously, and the question as to whether redundancy in teleoperation could be ensured by artificially shutting down and restoring the lines of one company at a time was satisfactorily verified. As a result, robotic teleoperation was possible without any major disruptions in communication quality that would affect the continuation of surgical operations, cause discomfort in surgical operations to the operator, or put restrictions on surgical operations. In addition, the remote surgeon hardly noticed any change due to the line switch, and there was almost no impact on the surgical task.

In other industries, studies have reported successful task completion such as remote automobile operation and manipulation using a redundant combination of multiple communication lines [25], and it has been confirmed that this type of system improves availability and contributes to reducing loss and delay compared to using only a single communication method. As in this study, it is very significant for telesurgery social implementation to strengthen the security and stability of communication by having multiple telecommunication entities supplementing information communication in the event of communication interruption. In addition, the involvement of multiple telecommunication carriers is expected to create price competition and improve economic efficiency.

Due in part to the recent pandemic, the search has begun to construct a new social system that fully utilizes digital and communication technology. Telesurgery using robots will create new medical values and educational methods and is expected to be implemented in society at an early date; the same is expected in many other fields as well.

### Limitations

Due to the limited validation time, the number of subjects was small and local and remote tasks could not be performed randomly. In addition, the subjects could not be verified randomly. Only Japanese robots were examined; examination using other robots, such as Da Vinci, was impossible for social and economic reasons. Only wired communication lines were considered. Wireless systems and internet connections such as 5G were not considered.

## Conclusion

It was shown that combining multiple communication lines guarantees communication redundancy and enables robotic teleoperation guidance with enhanced communication security.

## Supplementary Information

Below is the link to the electronic supplementary material.

Supplementary file1 (DOCX 19 kb)

Supplementary file2 (DOCX 17 kb)

Supplementary file3 (DOCX 17 kb)

Supplementary file4 (DOCX 18 kb)

## Data Availability

The datasets analyzed during the current study are available from the corresponding author upon reasonable request.
