# Supplementary material for: Ensuring communication redundancy and establishing a telementoring system for robotic telesurgery using multiple communication lines
Source: J Robot Surg. 2024 Jan 11;18(1):9. doi: 10.1007/s11701-023-01792-8 (PMC10784335; doi:10.1007/s11701-023-01792-8)
Supplement: Supplementary file 1 — Supplementary file1 (DOCX 19 kb) [file 11701_2023_1792_MOESM1_ESM.docx]

**Supplementary Table 1: Image Quality Score**

**Please answer the following questions about the surgical images of the robotic surgery you performed.**

On a scale of 1 to 5, with 5 being considered satisfactory for performing the procedure and 0 being considered completely unsuitable for performing the procedure, please rate the procedure.

**1. Clarity：How clear were the images from the robotic surgery?**

| It's not clear at all　　　　　　　　　　　　　　　　　　　　　　　　　　　Very clear | | | | |
| --- | --- | --- | --- | --- |
| 1 | 2 | 3 | 4 | 5 |

**2. Stereoscopic vision：Were the images of the robotic surgery in stereoscopic vision?**

| Was not stereoscopic vision at all　　　　　　　　　　　　　　Very stereoscopic vision | | | | |
| --- | --- | --- | --- | --- |
| 1 | 2 | 3 | 4 | 5 |

**3. Completeness：Was the robotic surgery screen complete?**

| Incomplete　　　　　　　　　　　　　　　　　　　　　　　　　　　　　　Complete | | | | |
| --- | --- | --- | --- | --- |
| 1 | 2 | 3 | 4 | 5 |

**4. Continuity：Were the robotic surgery screens continuous?**

| There's no continuity at all　　　　　　　　　　　　　　　　It's completely continuous | | | | |
| --- | --- | --- | --- | --- |
| 1 | 2 | 3 | 4 | 5 |

**5. Impact on the procedure：Were you able to perform the procedure with the images from this robotic surgery?**

| Could not perform at all　　　　　　　　　　　　　　　　　　Could be done perfectly | | | | |
| --- | --- | --- | --- | --- |
| 1 | 2 | 3 | 4 | 5 |

Total score:
