# Supplementary material for: Ensuring communication redundancy and establishing a telementoring system for robotic telesurgery using multiple communication lines
Source: J Robot Surg. 2024 Jan 11;18(1):9. doi: 10.1007/s11701-023-01792-8 (PMC10784335; doi:10.1007/s11701-023-01792-8)
Supplement: Supplementary file 2 — Supplementary file2 (DOCX 17 kb) [file 11701_2023_1792_MOESM2_ESM.docx]

Supplementary Table 2: Modified System usability scale (mSUS)

Please answer the following questions about the remote robotic surgical environment.

1: I don't think so at all 2: I don't think so 3: Neither 4: I think so 5: I strongly think so

| 1. I think I'll make sure I use this system often. | | | | |
| --- | --- | --- | --- | --- |
| 1 | 2 | 3 | 4 | 5 |
| 2. This system is simple and easy to use. | | | | |
| 1 | 2 | 3 | 4 | 5 |
| 3. This system is easy to use. | | | | |
| 1 | 2 | 3 | 4 | 5 |
| 4. I don't need technical support personnel to use this system. | | | | |
| 1 | 2 | 3 | 4 | 5 |
| 5. Many people will be able to use this system very soon. | | | | |
| 1 | 2 | 3 | 4 | 5 |
| 6. Gave me the confidence to use this system. | | | | |
| 1 | 2 | 3 | 4 | 5 |
| 7. This system is intuitive and easy to us. | | | | |
| 1 | 2 | 3 | 4 | 5 |
| 8. I found the functionality of this system very useful. | | | | |
| 1 | 2 | 3 | 4 | 5 |
| 9. This system was a great help in carrying out this task. | | | | |
| 1 | 2 | 3 | 4 | 5 |
