# Supplementary material for: Ensuring communication redundancy and establishing a telementoring system for robotic telesurgery using multiple communication lines
Source: J Robot Surg. 2024 Jan 11;18(1):9. doi: 10.1007/s11701-023-01792-8 (PMC10784335; doi:10.1007/s11701-023-01792-8)
Supplement: Supplementary file 3 — Supplementary file3 (DOCX 17 kb) [file 11701_2023_1792_MOESM3_ESM.docx]

Supplementary Table 3: Robot Usability Score

Please answer the following questions about the tele-robotic surgery environment.

1: I don't think so at all 2: I don't think so 3: Neither 4: I think so 5: I strongly think so

| 1. I was physically comfortable. | | | | |
| --- | --- | --- | --- | --- |
| 1 | 2 | 3 | 4 | 5 |
| 2. I had good hand control in this environment. | | | | |
| 1 | 2 | 3 | 4 | 5 |
| 3. I had good foot control in this environment. | | | | |
| 1 | 2 | 3 | 4 | 5 |
| 4. I had a good 3D field of view in this environment. | | | | |
| 1 | 2 | 3 | 4 | 5 |
| 5. I had no complaints and felt little stress in this environment. | | | | |
| 1 | 2 | 3 | 4 | 5 |
| 6. The robot moved smoothly. | | | | |
| 1 | 2 | 3 | 4 | 5 |
| 7. The robot did exactly what I wanted it to do. | | | | |
| １ | 2 | 3 | 4 | 5 |
| 8. I think I can actually perform surgery using this type of robotic surgery environment. | | | | |
| 1 | 2 | 3 | 4 | 5 |
| 9. If you answered 1-3 to question 8, please choose the reason from the following. If you have any other reason, please provide it as a free answer.  ・The image was rough. ・The robot didn't work the way I wanted it to.  ・My technique was inexperienced.  ・Other（ ） | | | | |

Name： Task No. １・２ Total score： /40
