# Supplementary material for: Ensuring communication redundancy and establishing a telementoring system for robotic telesurgery using multiple communication lines
Source: J Robot Surg. 2024 Jan 11;18(1):9. doi: 10.1007/s11701-023-01792-8 (PMC10784335; doi:10.1007/s11701-023-01792-8)
Supplement: Supplementary file 4 — Supplementary file4 (DOCX 18 kb) [file 11701_2023_1792_MOESM4_ESM.docx]

Supplementary Table 4: Questionnaire for changing line conditions

Name：

1. Did you notice any changes in the surgical environment during the procedure? Please check it.
   - I didn't notice. (→Please proceed to question 2.)
   - I noticed.

（→Please answer (1), (2), and (3) below in terms of what you noticed.）

1. Image Quality

　　　・There was a change in clarity.　□ Yes　　□ No

　　　　What is clarity?; No blur, in focus, smooth, clean, easy to see, fine

　　　・There was a change in three-dimensionality.　 □ Yes　　□ No

　　　　What is three-dimensional?; Three-dimensional appearance, perspective, depth, no distortion, no doubling

　　　・There was a change in integrity. 　□ Yes　　□ No

　　　　What is integrity?; No blurring or flickering, no unwanted images (lines or dots that are not really there)

　　　・There was a change in continuity. 　□ Yes　　□ No

　　　　What is continuous?; No time stoppage, no interruption of images, no missing images

1. Robot operability

　　　・There was a change in the robot's movements. 　 □ Yes　　□ No

　　　・The robot didn't work the way I wanted it to. 　 □ Yes　　□ No

1. Other (Please describe in detail)
2. Was your procedure inhibited by the surgical environment?

| Inhibited　　　　　　　　　　　　　　　　　　　　　　　　　　　　　Not inhibited | | | | |
| --- | --- | --- | --- | --- |
| 1 | 2 | 3 | 4 | 5 |

1. Can you actually perform surgery in a surgical environment like this one?

| I don’t think it’s possible. 　　　　　　　　　　　　　　　　I think it's possible. | | | | |
| --- | --- | --- | --- | --- |
| 1 | 2 | 3 | 4 | 5 |

1. Please feel free to describe any points you noticed about the overall environment of this surgery.
